# Supplementary material for: Epigenetic control of microglial mitochondrial immunity by KAT7 drives Alzheimer’s disease pathogenesis
Source: bioRxiv. 2026 Feb 20:2026.02.19.706884. Preprint. [Version 1] doi: 10.64898/2026.02.19.706884 (PMC12933571; doi:10.64898/2026.02.19.706884)
Supplement: Supplement 1 [file media-1.pdf]

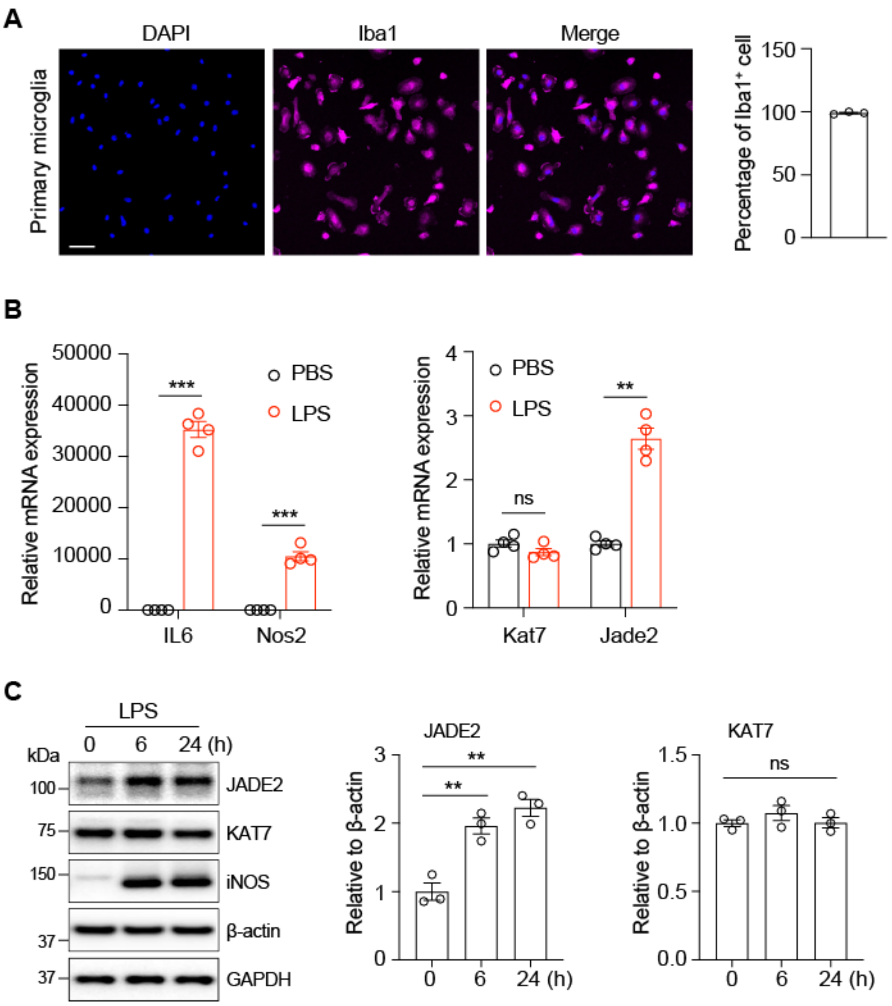

Suppl. Fig. 1. The expression of *Jade2* is upregulated in LPS-induced microglial activation. **A**, Representative images (left) and quantification (right) of Iba1 staining in cultured mouse primary microglia. Scale bar, 40 µm. n=3. **B**, qPCR analysis of indicated genes in primary microglia treated with LPS for 6 h. n=4, two-way ANOVA test. **C**, Western blot analysis of indicated protein in BV2 cells treated with LPS. Quantification was done by normalizing to β-actin (right). n=3, one-way ANOVA test. **p<0.01, ***p<0.001. Data are mean±SEM.

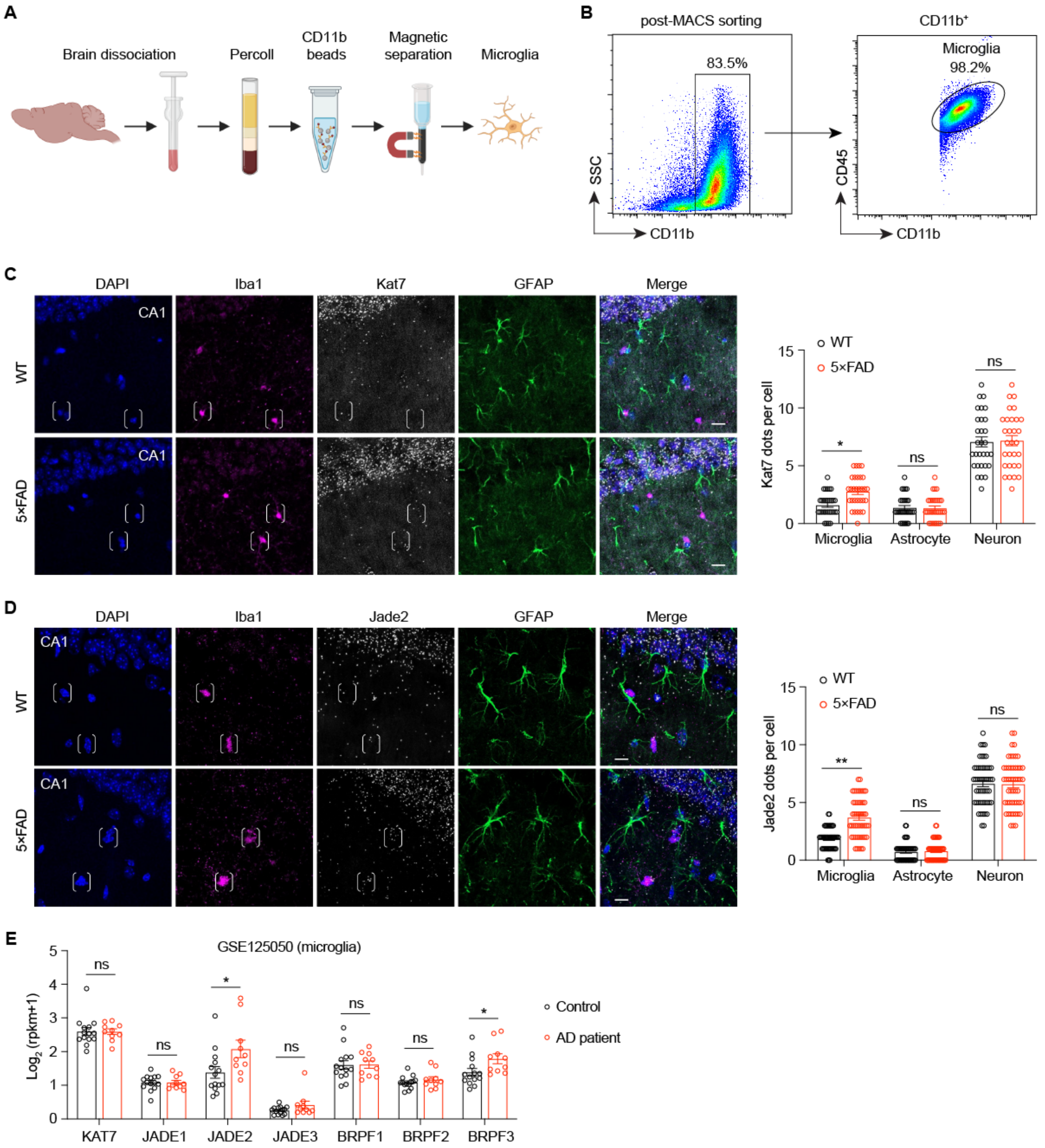

Suppl. Fig. 2. Expression of the KAT7 complex is elevated in microglia from both 5×FAD mouse model and human AD patients. **A**, Schematic diagram of microglia isolated from adult mouse brains. **B**, Representative FACS plots and gating strategy to check the purity of CD11b isolated microglia from the adult mouse brain. **C-D**, Left: Representative images of *Kat7* (**C**) or *Jade2* (**D**) RNAscope and its colocalization with Iba1- and GFAP-positive cells in hippocampal CA1 region of 7-month-old WT and 5×FAD mice. Scale bar, 10 µm. Right: Quantification in different cell types. n=30-50 cells from 3 mice per group. Two-way ANOVA test. **E**, Upregulation of JADE2 and BRPF3 in microglia from AD patients based on GSE125050 dataset. n= 14 control and 10 AD. Unpaired student's *t*-test. *p<0.05, **p<0.01. ns, nonsignificant. Data are mean±SEM.

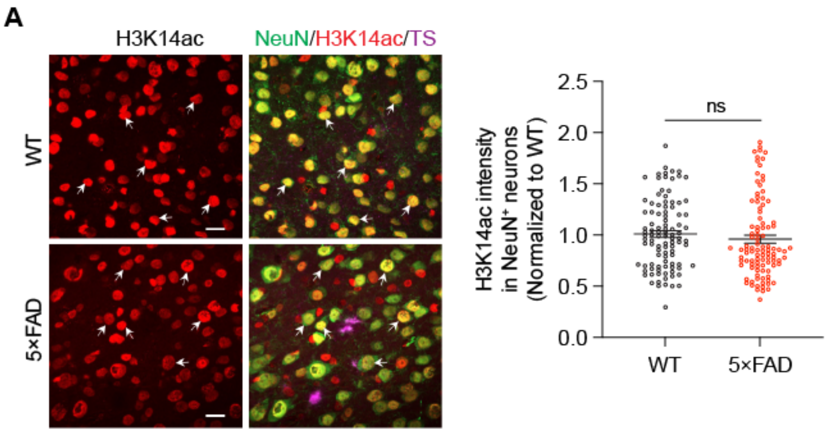

Suppl. Fig. 3. H3K14ac levels are not changed in neurons or astrocytes of 5×FAD mice. **A**, Left: Representative images of H3K14ac co-stained with neurons (NeuN) in the cortex region of 6-month-old WT and 5×FAD mice. Scale bar, 20 µm. Right: Quantification of H3K14ac intensity in neurons (n=100 cells from 3 mice per group). White arrowheads indicate H3K14ac in neurons. Mann-Whitney test. **B**, Left: Representative images of H3K14ac co-stained with astrocytes (GFAP) in the cortex region of 6-month-old WT and 5×FAD mice. Scale bar, 20 µm. Right: Quantification of H3K14ac intensity in astrocytes (n=100 cells from 3 mice per group). White arrowheads indicate H3K14ac in astrocytes. Mann-Whitney test. ns, nonsignificant. Data are mean±SEM.

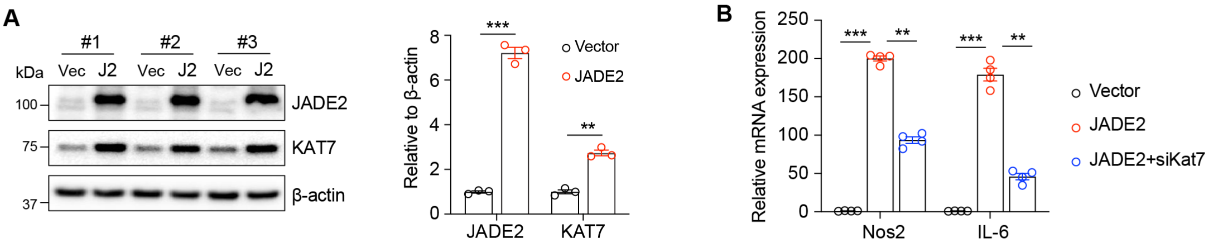

Suppl. Fig. 4. JADE2 overexpression enhances expression of pro-inflammatory factors. **A**, Western blot analysis of JADE2 overexpression in BV2 cells. Quantification was done by normalizing to β-actin (right). n=3. **B**, qPCR analysis of *Nos2* and *Il-6* levels in BV2 cells. n=4. **p<0.01, ***p<0.001. Two-way ANOVA test. Data are mean±SEM.

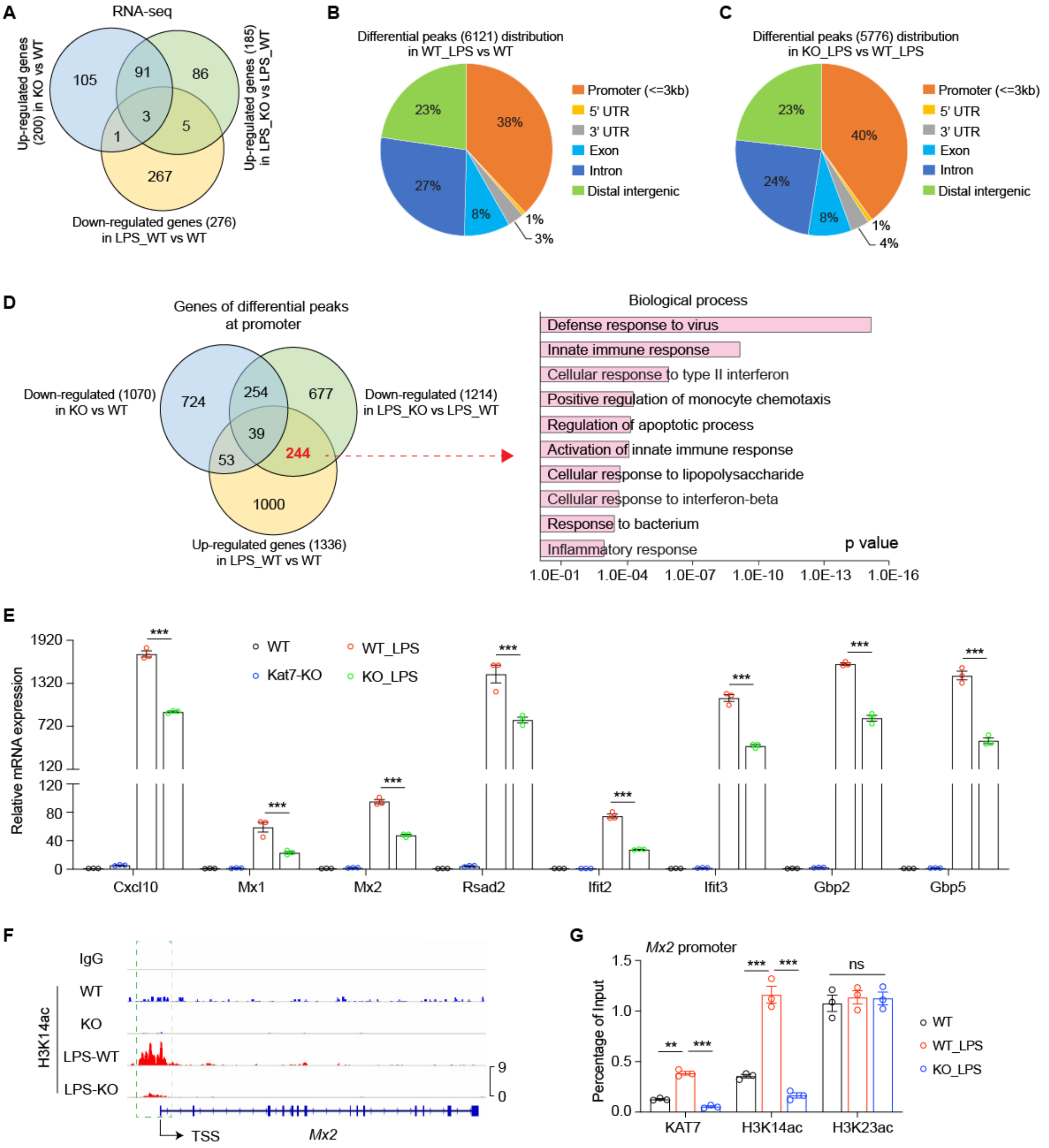

Suppl. Fig. 5. Analysis and validation of RNA-seq and CUT&Tag data. **A**, Venn diagram of overlapping genes in RNA-seq. **B**, Genomic distribution of differential H3K14ac-binding peaks in WT_LPS vs WT. **C**, Genomic distribution of differential H3K14ac-binding peaks in KO_LPS vs WT_LPS. **D**, Left: Venn diagram of overlapped genes of differential peaks at promoter among downregulated in KO vs WT, downregulated in KO_LPS vs WT_LPS, and upregulated in WT_LPS vs WT. Right: GO pathway analysis of the 244 overlapped genes. **E**, qPCR analysis showed the expression of the indicated genes in BV2 microglia with or without LPS treatment. n=3. **F**, Representative CUT&Tag tracks of H3K14ac in *Mx2*. Green box indicated proximal promoter. TSS, transcriptional start site. **G**, qChIP analysis of *Mx2* promoter using the indicated antibodies in BV2 cells. n=3. $^{**}p<0.01$, $^{***}p<0.001$. Two-way ANOVA test. Data are mean±SEM.

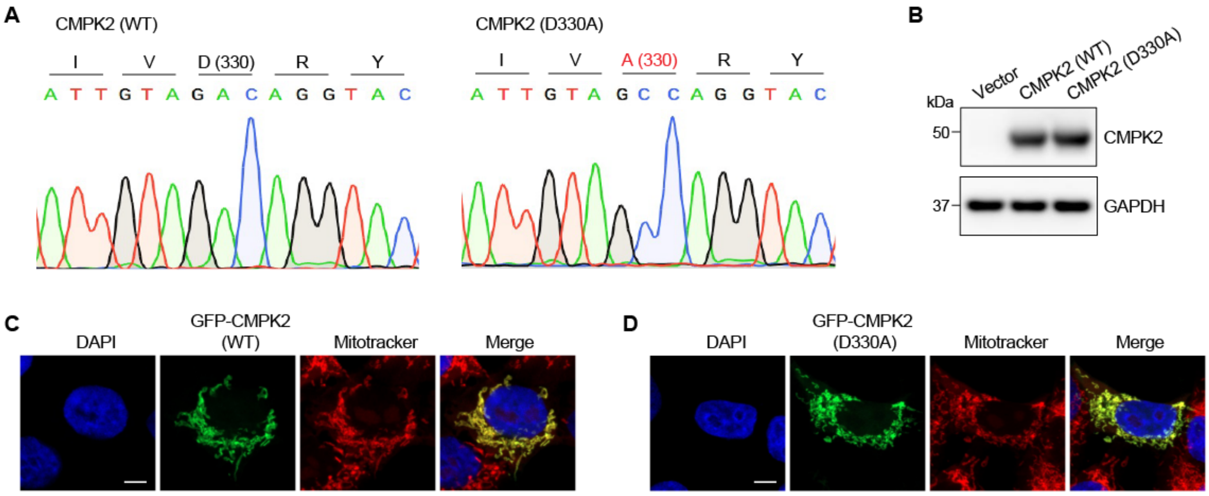

Suppl. Fig. 6. The CMPK2 mutant is still expressed in mitochondria. A, Sanger sequencing validated the CMPK2 mutant (D330A) sequence. **B**, Western blot confirmed the infection efficiency of CMPK2 WT and mutant lentiviruses in BV2 microglia. **C-D**, Representative images of GFP-CMPK2 (**C**) or GFP-CMPK2-D330A (**D**) co-stained with Mitotracker in HEK-293T cells.

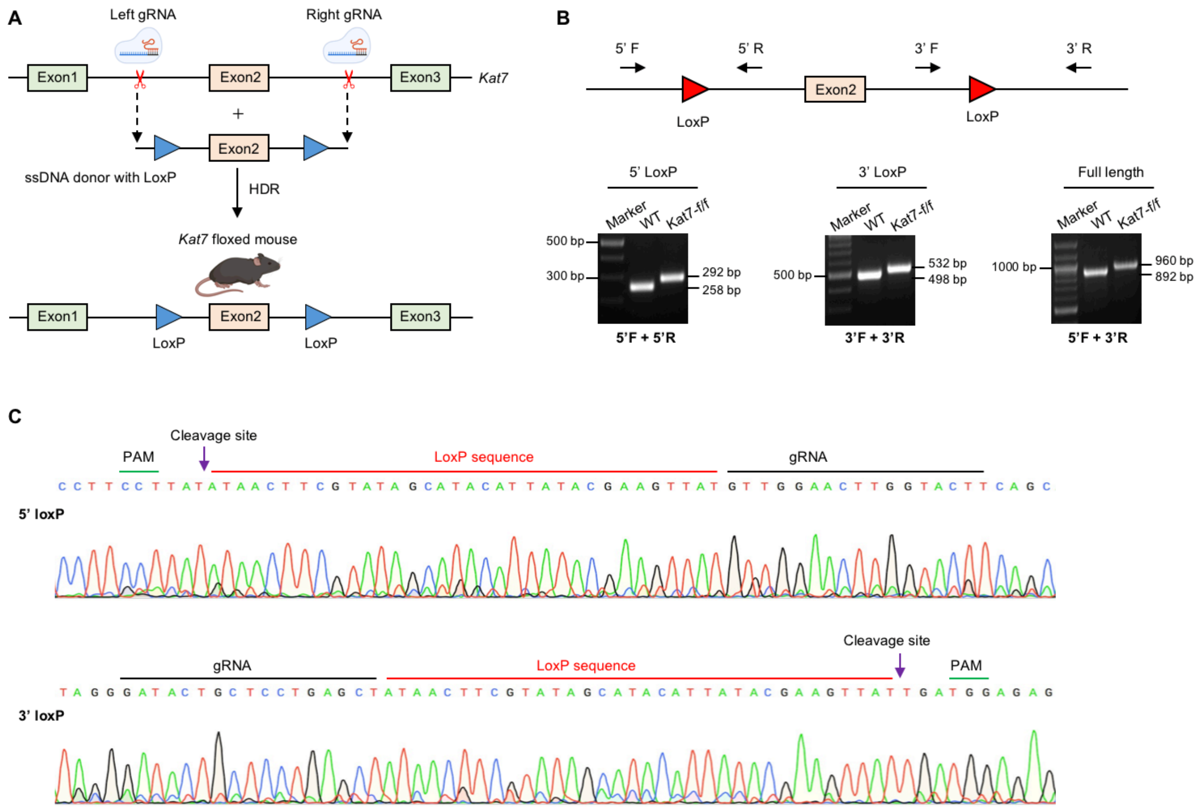

Suppl. Fig. 7. Generation of *Kat7*-floxed mice. **A**, Scheme of *Kat7*-floxed mice generation using CRISPR-Cas9 method. HDR, homology-directed repair. **B-C**, Validation of *Kat7*-floxed mice by mouse tail genotyping (**B**) and Sanger sequencing (**C**).

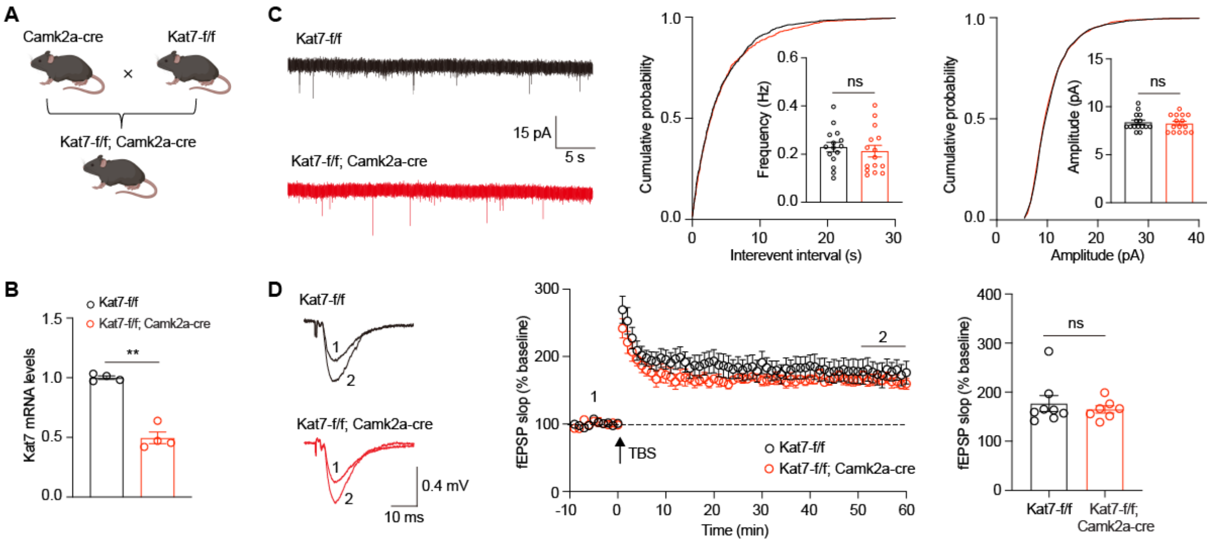

Suppl. Fig. 8. Deletion of *Kat7* in excitatory neurons does not alter synaptic function in mice. **A**, *Kat7* floxed mice crossed with Camk2a-cre mice to obtain the *Kat7*$^{\text{Camk2a-cKO}}$ mice. **B**, qPCR analysis of total hippocampal mRNA from *Kat7*$^{\text{f/f}}$ and *Kat7*$^{\text{Camk2a-cKO}}$ mice. n=4 mice per group. **C**, Representative traces (left) and quantification of frequency (middle) and amplitude (right) of mEPSC in hippocampal CA1 pyramidal neurons. n=15 cells from 3 mice per group. **D**, TBS-induced LTP at Schaffer collateral to CA1 synapses. Arrow indicates LTP induction. n=7-8 slices from 4 mice per group. Mann-Whitney test. ns, nonsignificant. Data are mean± SEM.

Table S1. The information for formalin-fixed paraffin embedded (FFPE) tissue slides of frontal cortex from control and AD patients.

Case	Patient GUID	Age	Gender	Tissue
Control 1	BRC2052	79	Male	Middle frontal gyrus
Control 2	BRC2151	72	Male	Middle frontal gyrus
Control 3	BRC2234	68	Female	Middle frontal gyrus
Control 4	BRC2317	65	Female	Middle frontal gyrus
AD 1	BRC2609	68	Male	Middle frontal gyrus
AD 2	BRC2636	80	Female	Middle frontal gyrus
AD 3	BRC2718	70	Male	Middle frontal gyrus
AD 4	BRC2641	72	Female	Middle frontal gyrus

Table S2. Mouse genotyping primers

Mouse strain	Strand	Sequence
Kat7-floxed mice	5'F	AGTACAGGTGGTTTGGTTGT
	5'R	AATCGGAATCTTCGGTTCCA
	3'F	CACAGTGGACAGTGGTGTCA
	3'R	TCAGCAGCTGCCTTACACTT
Cx3cr1-Cre^{ER}	F (common)	AAGACTCACGTGGACCTGCT
	R (WT)	AGGATGTTGACTTCCGAGTTG
	R (Cre^{ER})	CGGTTATTCAACTTGCACCA
Camk2a-Cre	F	GTTCTCCGTTTGCACTCAGG
	R	CAGGTTCTTGCGAACCTCAT
5×FAD	F (common)	ACCCCCATGTCAGAGTTCCT
	R (WT)	TATACAACCTTGGGGGATGG
	R (mutant)	CGGGCCTCTTCGCTATTAC

Table S3. Real-time qPCR primers

Mouse gene name	Strand	Sequence
Kat7	F	TGCAGGCAGTAGTTCAGATGG
	R	CAGGGCTGGAATCTTGGGAA
Jade1	F	ATGTCTGCCAGTCACCTGATGG
	R	ACGACATAGCCAACTGCCCTCT
Jade2	F	CCAAGACTGACGAGGTGGACAA
	R	CCGTCTTGTCACCATGTAGCAC
Jade3	F	GTCTCCAGACAGTGAAGAAGGG
	R	GCACAGCCAACTACCTTCTGGA
Brpf1	F	CCAAGAGAAGGACACAGGCAAC
	R	GTAGCGGTAAGCCTCCAAGTTC
Brpf2	F	GGAGGCTTTGAAGATGAGGCTG
	R	GGTGGTTCTGAGTTAGTCTCCG

Brpf3	F	CTCATCCGCAAAAGGGAGAAGC
	R	TCCAGAGTCGTCCTCAACAGGA
Nos2	F	CAGCTGGGCTGTACAAACCTT
	R	CATTGGAAGTGAAGCGTTTCG
Il-6	F	CTGCAAGAGACTTCCATCCAG
	R	AGTGGTATAGACAGGTCTGTTGG
Cmpk2	F	AACTCTGCGGTGTTCCAAGACC
	R	GGAACTTCCCTTTCTGGACCTC
Cxcl10	F	ATCATCCCTGCGAGCCTATCCT
	R	GACCTTTTTTGGCTAAACGCTTTC
Rsad2	F	GGAAGGTTTTCCAGTGCCTCCT
	R	ACAGGACACCTCTTTGTGACGC
Ifit2	F	CGAACTACCGTCTGGATGACTG
	R	CTTCAACCAGCGCCATTGCTTG
Ifit3	F	GCTCAGGCTTACGTTGACAAGG
	R	CTTTAGGCGTGTCCATCCTTCC
Gbp2	F	AGATGCCCACAGAAACCCTCCA
	R	AAGGCATCTCGCTTGGCTACCA
Gbp5	F	GAACGCCAAAGAAACAGTGAGCC
	R	CTTCCTGGATGCGAATAGCCTC
Mx1	F	TGGACATTGCTACCACAGAGGC
	R	TTGCCTTCAGCACCTCTGTCCA
Mx2	F	ACCAGAGTGCAAGTGAGGAGCT
	R	GTACTAGGGCAGTGATGTCCTG
β-actin	F	GGCTGTATTCCCCTCCATCG
	R	CCAGTTGGTAACAATGCCATGT
Gapdh	F	GGGTGTGAACCACGAGAAATA
	R	CTGTGGTCATGAGCCCTTC

Table S4. mtDNA qPCR primers

**Gene name**	**Strand**	**Sequence**
Mouse *D-loop*	F	AATCTACCATCCTCCGTGAAACC
	R	TCAGTTTAGCTACCCCCAAGTTTAA
Mouse *Nd4*	F	AACGGATCCACAGCCGTA
	R	AGTCCTCGGGCCATGATT
Mouse *B2m*	F	ATGGGAAGCCGAACATACTG
	R	CAGTCTCAGTGGG GGTGAAT
Mouse *Tert*	F	TAGCTCATGTGTCAAGACCCTCTT
	R	GCCAGCACGTTTCTCTCGTT

Table S5. ChIP-qPCR primers

Gene name	Strand	Sequence
Mouse *Cmpk2* promoter	F	ATCCCACGTGAGCAAAGGG
	R	CCTGAAGCGTATTTGGGCAG
Mouse *Mx2* promoter	F	AGTTCCCCAAGAACCAGAGAAATG
	R	ATTATGATGGGAAAGGCAGGTTCA
